# Ion transport and current rectification in a charged conical nanopore filled with viscoelastic fluids

**DOI:** 10.1038/s41598-022-06079-w

**Published:** 2022-02-15

**Authors:** Mohit Trivedi, Neelkanth Nirmalkar

**Affiliations:** Department of Chemical Engineering, Indian Institute of Technology Ropar, Rupnagar, 140001 India

**Keywords:** Engineering, Chemical engineering

## Abstract

The ionic current rectification (ICR) is a non-linear current-voltage response upon switching the polarity of the potential across nanopore which is similar to the I–V response in the semiconductor diode. The ICR phenomenon finds several potential applications in micro/nano-fluidics (e.g., Bio-sensors and Lab-on-Chip applications). From a biological application viewpoint, most biological fluids (e.g., blood, saliva, mucus, etc.) exhibit non-Newtonian visco-elastic behavior; their rheological properties differ from Newtonian fluids. Therefore, the resultant flow-field should show an additional dependence on the rheological material properties of viscoelastic fluids such as fluid relaxation time $(\lambda )$ and fluid extensibility $(\varepsilon )$. Despite numerous potential applications, the comprehensive investigation of the viscoelastic behavior of the fluid on ionic concentration profile and ICR phenomena has not been attempted. ICR phenomena occur when the length scale and Debye layer thickness approaches to the same order. Therefore, this work extensively investigates the effect of visco-elasticity on the flow and ionic mass transfer along with the ICR phenomena in a single conical nanopore. The Poisson–Nernst–Planck (P–N–P) model coupled with momentum equations have been solved for a wide range of conditions such as, Deborah number, $1\le De \le 100$, Debye length parameter, $1\le \kappa R_t \le 50$, fluid extensibility parameter, $0.05\le \varepsilon \le 0.25$, applied electric potential, $-40\le V \le 40$, and surface charge density $\sigma = -10$ and $-50$. Limited results for Newtonian fluid ($De = 0$, and $\varepsilon = 0$) have also been shown in order to demonstrate the effectiveness of non-Newtonian fluid behaviour over the Newtonian fluid behaviour. Four distinct novel characteristics of electro-osmotic flow (EOF) in a conical nanopore have been investigated here, namely (1) detailed structure of flow field and velocity distribution in viscoelastic fluids (2) influence of Deborah number and fluid extensibility parameter on ionic current rectification (ICR) (3) volumetric flow rate calculation as a function of Deborah number and fluid extensibility parameter (4) effect of viscoelastic parameters on concentration distribution of ions in the nanopore. At high applied voltage, both the extensibility parameter and Deborah number facilitate the ICR phenomena. In addition, the ICR phenomena are observed to be more pronounced at low values of $\kappa R_t$ than the high values of $\kappa R_t$. This effect is due to the overlapping of the electric double layer at low values of $\kappa R_t$.

## Introduction

Over the years, synthetic nanopores have attracted significant research attention due to their relevance and potential application in bio-sensing devices, DNA sequencing^1^, micro-nano pumping devices^2^, mixing applications^3^, and polymer translocation^4–9^. A detailed understanding of flow and ionic transport in these nanopores is imperative not only in the investigation and interpretation of physiological processes/mechanisms in living beings but also in the development of smart sensing devices based on the electrochemical properties of the flow system^10^. The ionic current through a conical nanopore at a negative applied voltage bias differs from that at a positive applied voltage bias, implying that ion transport is preferential in one direction. It is widely noticed that when the Debye length is comparable to the diameter of a conical nanopore, the ionic current rectification (ICR) phenomena occurs due to ionic concentration imbalance across the electrical double layer^11^. Thus, the ICR phenomenon in a nanopore is analogous to an asymmetric diode-like ionic current-voltage behaviour. In addition, the ionic selectivity and ICR properties of the nanopore can also be utilized to detect and quantify the characteristics of the flow field, and the ionic concentration distribution^12^. Therefore, the ICR phenomena are fundamentally related to the viscous and elastic behavior of the fluid and the electrical properties (relative permittivity, ionic diffusivity, and the valency of the cation and anions) in the fluid^13,14^. In this work, elastic effects on ICR phone have been investigated in a single conical nanopore. The Poisson–Nernst–Planck (P–N–P) model coupled with momentum equations have been solved using finite volume method. Five distinct novel features of electro-osmotic flow (EOF) in a conical nanopore have been investigated here, namely (1) the effects of the memory of visco-elastic fluid and extensibility has been investigated in terms of detailed structure of flow field and velocity distribution (2) analysis of visco-elastic effects on volumetric flow rate (3) detailed findings on the influence of the Debye length, Deborah number and fluid extensibility parameter on ionic current rectification (ICR) and discussions on the applied potential over the ionic current behavior (4) volumetric flow rate calculation and analysis as a function of Deborah number and fluid extensibility parameter (5) Effects of visco-elastic parameters on ionic concentration distribution. In a pioneer study of Wei et al.^15^, the ICR phenomena have been observed for the flow of a KCl solution in a silica nanopore.

**Figure 1:**
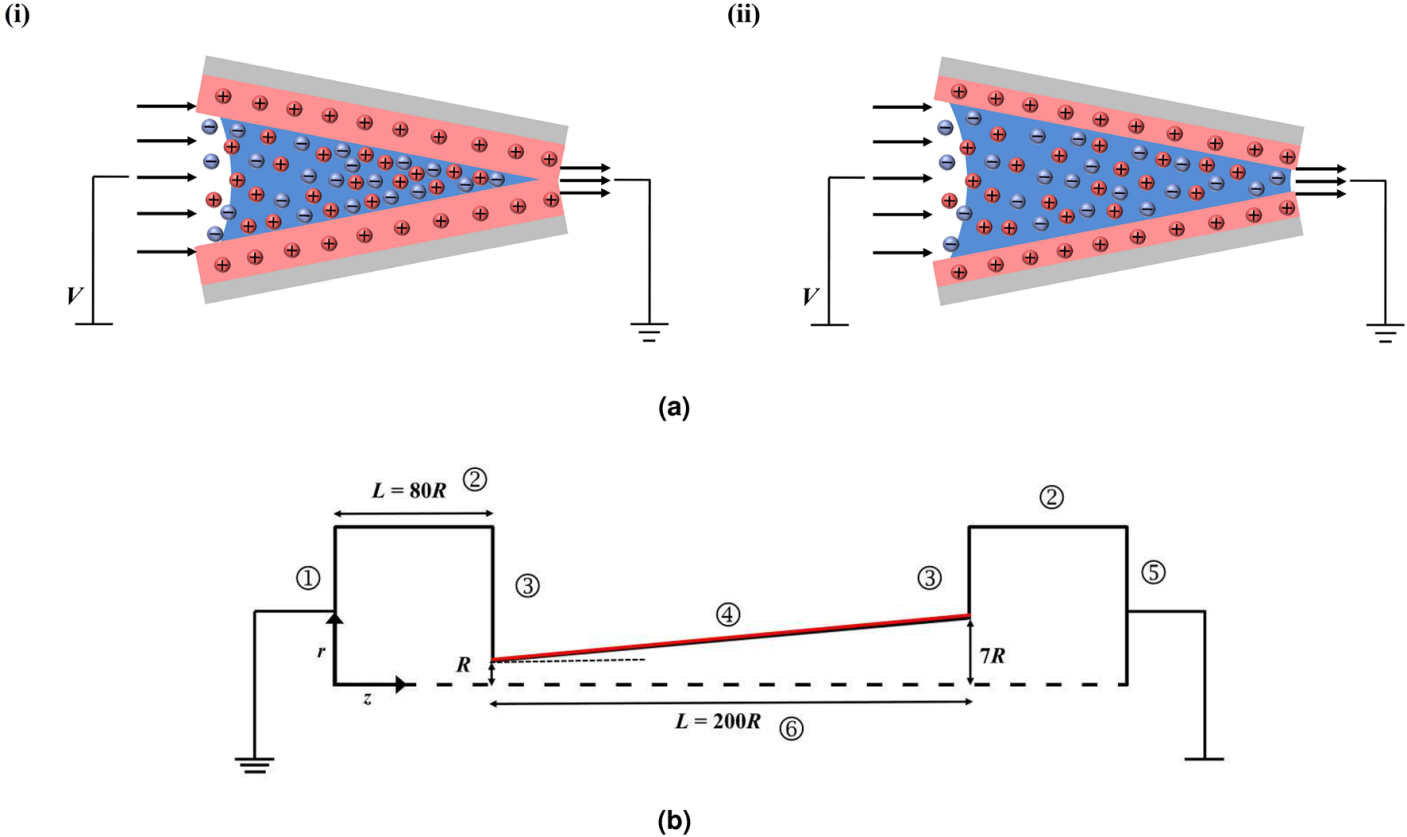
**(a)** Conical nanopore with a forward potential bias. The electric double layer is highlighted with the red shading. The blue shading represents the bulk solution. (i) When electric double layers are thick and interaction occurs at the nanopore tip (ii) When they are thin and there is no interaction. Dimensions are not to scale. **(b)** Schematic diagram of computational domain.

Several studies^11,16–21^ have since then reported the ICR in synthetic nanopores. It was concluded that the resistance to the ionic current depends on the flow direction, surface charge, and nonuniformity of cross-section of the nanopore^11,18,22,23^. This feature of nanopores can potentially be utilized for separation processes^24^ or lab-on-a-chip applications^25^. The nanopores can be both symmetric or asymmetric in shape. Example of symmetric shapes are cylindrical nanopores while a conical nanopore is known as a asymmetrical shape. When such nanopore surfaces are given a finite charge and introduced to an ionic fluid, an immobile and stationary electric double layer (EDL) is developed over the surface. The thickness of this layer is based on several factors like the charge density of the pore-wall, the valency and concentration of the ions present in the fluid etc. Moreover, these EDLs formed at nanopore walls can be carefully tuned to vary the thickness of the EDL. As the value of EDL thickness becomes equivalent to the nonpores radius the flow through the nanopore is suppressed because of the blockage in the cylindrical nanopore. This property of the nanopore could be very interesting for an asymmetric shaped nanopore or a conical nanopore. The gradual increase/decrease in the flow area coupled with the varying EDL thickness can act as a valve-like nature for a conical nanopore. Moreover, on the contrary to the cylindrical nanopore, for a given positive voltage bias, a conical nanopore will exhibit higher flow-rate and ionic current than that at a negative potential bias of the same magnitude. These nano-scale valves can be used to design and control the value of the flow and ionic current passing through them in a micro-fluidic pumping devices or in Lab-in-Chip applications. Therefore, since the ionic current flowing through the nanopore is modulated and rectified due to the asymmetric (diverging and/or converging) shape of the nanopore, it holds an analogy with the function of an electrical transistor^26^ too. A positive applied potential bias in a conical nanopore offers a high ionic current under electro-osmotic flow (EOF), whereas a negative applied potential bias reduces the ionic current significantly, suggesting higher electrical resistivity in the nanopore for negative or backward applied potential bias^27^. While the ICR phenomenon is quantified in terms of the net rate of ion transfer or the ionic current, it also yields an effective electro-osmotic flow rectification (EFR)^28^. EFR property of a nanopore can be utilized to design nano-micro scale pumping devices^29^. Some studies^13,30^ present excellent comparisons between the experiment and the analytical solutions based on Poisson-Nernst-Planck (PNP) equations.

**Figure 2:**
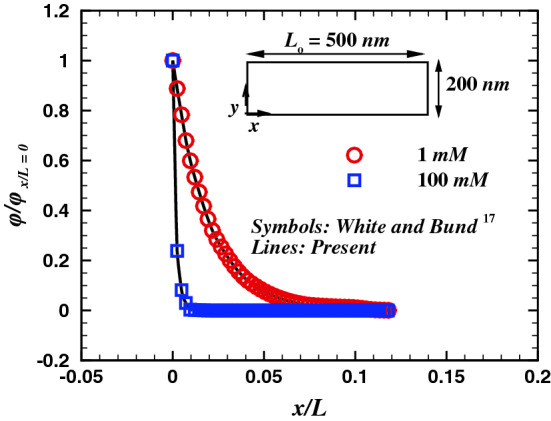
Comparison of the variation of cross-sectional potential profile with White and Bund^17^ at ${\rho _s} = -1 \quad mC/{m^2}$.

These results have consolidated that if the diameter of the pore is larger than 10% of the Debye length^31,32^ the applicability of the continuum hypothesis is still valid. Therefore, numerical studies have also been performed for the flow and ion transfer through a nanopore over recent years. Though, few of these studies have incorporated the effect of the electro-osmotic flow induced from the interaction of the charge density and the applied electric field via the PNP model. The findings of such studies are limited to some special cases^13,16,33^. It has been observed from these studies that the applied electric field in conjunction with the surface charge on the nanopore significantly influence the flow field and consequently the ionic current distribution in the nanopore^17,34^. Furthermore, since most of the biological fluids, such as mucus, saliva, blood and other lipids^35,36^ exhibit viscoelastic flow behavior. Therefore, their rheological flow properties exhibit additional features, i.e., the memory effect and principal stress distribution in addition to shear-dependent viscosity^37^. Hence, the electro-osmotic flow and the ICR phenomena of such fluids are expected to differ from the conventional generalized Newtonian fluid (GNF) behavior. Some recent studies on micro/nanofluidics have considered non-Newtonian flow behavior, e.g., an analytical solution of the EOF of power-law fluids in a rectangular micro-channel has been investigated by Das and Chakraborty^38^. They have observed that the non-Newtonian shear-thinning nature facilitates the concentration distribution, whereas it also has insignificant influence over the concentration distribution. Zimmerman et al.^39^ have numerically studied the electro-osmotic flow in a T-junction for Carreu-Yasuda fluids, and the resulting end wall pressure profile has been found to be dependent on the relaxation time and power-law index. The EOF in rectangular and cylindrical tubes for various (Power-law fluids, Bingham plastic fluids, and Eyring fluids) non-Newtonian models have been studied by Berli and Olivares^40^, it has been concluded that the effect of non-Newtonian rheology is confined to only the diffusive ionic flux through micro-channel. Analytical results for power-law flow behavior and power-law index, *n* has been investigated by Zhao and Yang^41^, and it has been shown that increasing the electrokinetic parameter $\kappa H$ or decreasing the power-law index *n* can lead to an increase in the volumetric flow rate *Q* for the electro-osmotic flow of power-law fluids. A few notable studies on viscoelastic flow in micro and nano-slits/circular cross-section have also been presented by Li et al.^42^, and Wang et al.^43^ for Maxwell fluids, while Mei et al.^44^, Chen et al.^45^ and Park and Lee^46^ have investigated Phan–Thien–Tanner (PTT) fluids. While no prior studies on the effect of visco-elasticity on ICR phenomena have been reported yet. Hence, the present study is dedicated to investigate the flow of simplified Phan–Thien–Tanner fluids through a conical nanopore and the ICR phenomena. The results are presented in the form of streamlines, velocity contours, velocity profiles, and current vs. potential (*I* vs *V*) plots.

## Problem description

The steady-state electro-osmotic flow of visco-elastic (sPTT) binary electrolyte has been considered in a conical nanopore (Fig. 1a) with a tip Radius $R_t$ and axial length $H = 200R_t$ and divergent angle^47^ $\alpha = 1.432^\circ$. The flow field has been considered to be 2-D axi-symmetric. Since the flow, concentration, and potential field have been expected to be symmetric along the $\theta$ direction. Therefore, a thin 2*D* slice of the domain is considered here in *r-z* co-ordinate frame for the numerical study (Fig. 1b). The nanopore sizes typically range from 2 to 150 nm^48^, and the influences of gravitational force can be neglected in such small size ranges, therefore the gravitational force has been considered to have no effect on flow, concentration, and potential fields. The effect of variation in cone angle has been also discussed in the terms of radial velocity profiles and *I* vs *V* curves in this study and the corresponding results (Figs. 7 and 11). Due to the interaction of the negatively charged pore surface and the counter charged (positive) ions, a layer of positively charged ions forms on the charged wall surface, followed by a relatively thick convective layer of counter-charged ions known as the electric double layer (EDL). At the nanopore surface, the positive ions are concentrated near the charged wall, and the co-ions (negative) remain mostly close to the core of the flow. It is also considered that the ionic concentration is very low in the solution (i.e., dilute electrolyte). Therefore, the Boltzmann equilibrium can be considered. EDL thickness is often given by $\lambda _D = 1/\kappa$, which can be scaled by the length scale, $R_t$ as $\kappa R_t$. An electric field *E* is applied in the *z*-direction by applying a potential bias *V* across the nanopore. The ICR phenomena occur when the electric double layer overlaps at the tip (convergent end) of the nanopore. The electric double layer does not overlap when $\kappa R_t$ is high and consequently, the ion migration does not gets affected by the double layer. Therefore, the ions at the core of the fluid remain solely driven by the applied potential bias. As the value of the $\kappa R_t$ decreases (i.e., the thickness of the double layer increases), the ionic current through the pore gets dampened by the resisting forces, arising due to the ionic charge density near the tip of the nanopore. This behavior is analogous with the characteristics of a p-n type electric transistor^26^. Therefore, at a forward potential bias (i.e., the higher potential at the convergent end of the pore), the flow of cations is directed towards the divergent end of the pore. The flow configuration of ions in forward potential bias assures that the double layers do not overlap, and thus the resulting electrical resistance reduces significantly. At the reverse bias, the cations are forced to flow towards the convergent end of the nanopore, and these cations get accumulated at the end of the nanopore due to the overlapped electric double layer. Thus, in order to delineate the effect of the interaction of the electric double layer and applied electric field at the tip of the nanopore, the coupled potential, concentration, and velocity fields are solved using Poisson–Nernst–Planck (P–N–P) model.

**Figure 3:**
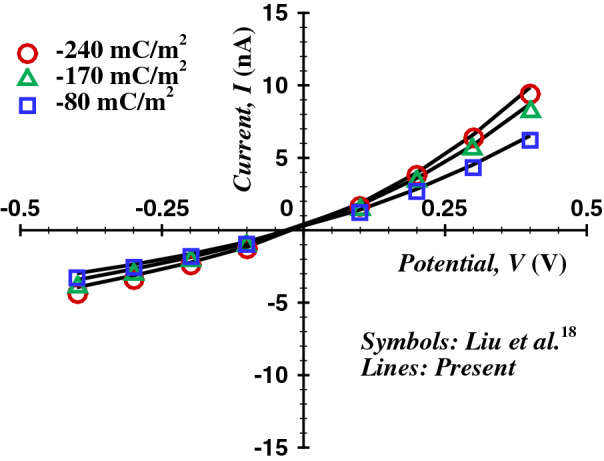
Comparison of the variation in average current with applied voltage for a bulk concentration of KCl at 0.05 M with Liu et al.^18^.

### Governing equations

The flow induced by applied potential difference is given by the equations of continuity and momentum and it is written as follows:

1 $$\begin{aligned}{}&\nabla \cdot u=0 \end{aligned}$$

2 $$\begin{aligned}{}&-\nabla p+\nabla \cdot \tau + \rho _e E = 0 \end{aligned}$$

the potential field is given by Poisson’s equation as follows:

3 $$\begin{aligned} \begin{aligned}{}&{\nabla }^2 \psi = -{\frac{\rho _e}{\varepsilon _p}} \end{aligned} \end{aligned}$$

where the space charge density $\rho _e$ is defined as:

4 $$\begin{aligned} \begin{aligned}{}&{\rho _e}=F\sum _{i=1}^{2} {z_{i}} {c_{i}} \end{aligned} \end{aligned}$$

where: $z_i$ and $c_i$ are the valance and concentration of $i_{th}$ species respectively. The externally applied electric field is expressed in terms of the potential gradient as follows:

5 $$\begin{aligned} \begin{aligned}{}&E = -\nabla \psi \end{aligned} \end{aligned}$$

Similarly, the ionic flux balance across the nanopore is expressed by the Nernst–Planck equation:

6 $$\begin{aligned} \nabla \cdot N_i = 0, \quad i=1, 2 \end{aligned}$$

where

7 $$\begin{aligned} N_i = \varvec{u} c_{i}-D_{i} \nabla c_{i}-{\frac{e z_{i} D_{i} c_{i}}{k_B T}} \nabla _{i} \psi \quad i=1, 2 \end{aligned}$$

**Figure 4:**
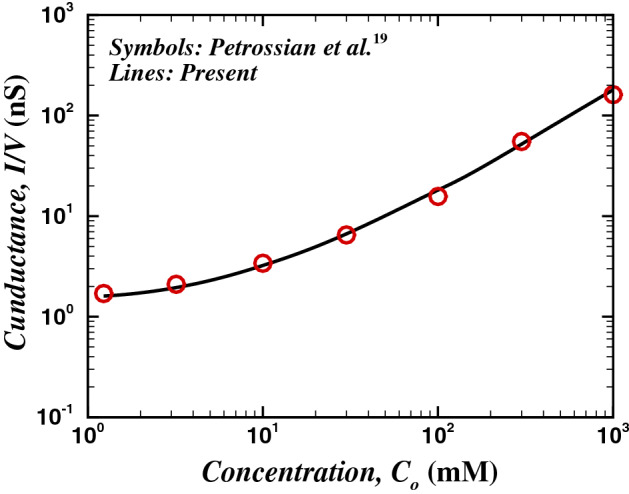
Comparison of the variation in average conductance (*I*/*V*) with bulk average concentration with Petrossian et al.^19^.

The constitutive equation of the *sPTT* model^49,50^ has been given by:

8 $$\begin{aligned} f\left( \tau _{\text {kk}}\right) \tau +\lambda \overset{\nabla }{\tau } =2 \eta _P \mathbf{D } \end{aligned}$$

where $\eta _P$ is the polymeric viscosity coefficient, $\lambda$ is the relaxation time of the viscoelastic fluid and $\mathbf{D} = (\nabla u^T+ \nabla u)/2$ is the rate of deformation tensor, the $f (\tau _{kk})$ is called the stress coefficient and can be defined as:

9 $$\begin{aligned} f\left( \tau _{\rm{kk}}\right) =1+(\varepsilon \lambda / \eta _P) \tau _{\rm{kk}} \end{aligned}$$

where, $\tau _{kk}$ denotes the trace of the extra stress tensor and $\varepsilon$ is known as the PTT parameter. It governs the extensibility and elongation property of the PTT fluid^49,50^. The $\overset{\nabla }{\tau }$ is the upper convective derivative of the polymeric tensor and can be defined as:

10 $$\begin{aligned} \overset{\nabla }{\tau }=\frac{{\rm {D}} \tau }{{\rm {D}} Y}-\nabla u^{T} \cdot \tau -\tau . \nabla u \end{aligned}$$

Eqs. (1–7) have been rendered dimensionless using the corresponding scaling variables as listed in Table 1. They are written in their respective dimensionless forms as follows:

**Table 1:** The scaling variables and their definition.

Scale	Symbol	Definition
Length scale	$R_t$	$R_t$
Velocity scale	$U_o$	$\frac{\varepsilon _p {k_B^{2}}{T^{2}}}{\eta _P R_t {e^2}{z^2}}$
Potential scale	$V_T$	$\frac{{k_B} T }{ez}$
Ionic concentration scale	$C_o$	$C_o$
Ionic current	$I_o$	${F{C_o}{U_o}{{R_t}^2}}$

11 $$\begin{aligned}{}&\nabla ^* . u^* = 0 \end{aligned}$$

12 $$\begin{aligned}{}&-\nabla ^* p^*+\nabla ^* \cdot \tau ^*+\frac{(\kappa R_t)^2}{2} \sum _{i=1}^{{\rm 2}} {z_{i}} {c_{i}}^*\nabla ^* \psi ^* = 0 \end{aligned}$$

13 $$\begin{aligned}{}&{{\nabla }^2}^* \psi ^* = \frac{(\kappa R_t)^2}{2} \sum _{i=1}^{2} {z_{i}} {c_{i}}^* \end{aligned}$$

14 $$\begin{aligned}{}&\nabla ^{*} \cdot N^{*}=0 \end{aligned}$$

15 $$\begin{aligned}{}&N^{*} = \varvec{u}^{*} c_{i}^{*}-D_{i}^{*} \nabla ^{*} c_{i}^{*}-z_{i} D_{i}^{*} \nabla ^{*} \psi ^{*} \quad i=1, 2 \end{aligned}$$

**Figure 5:**
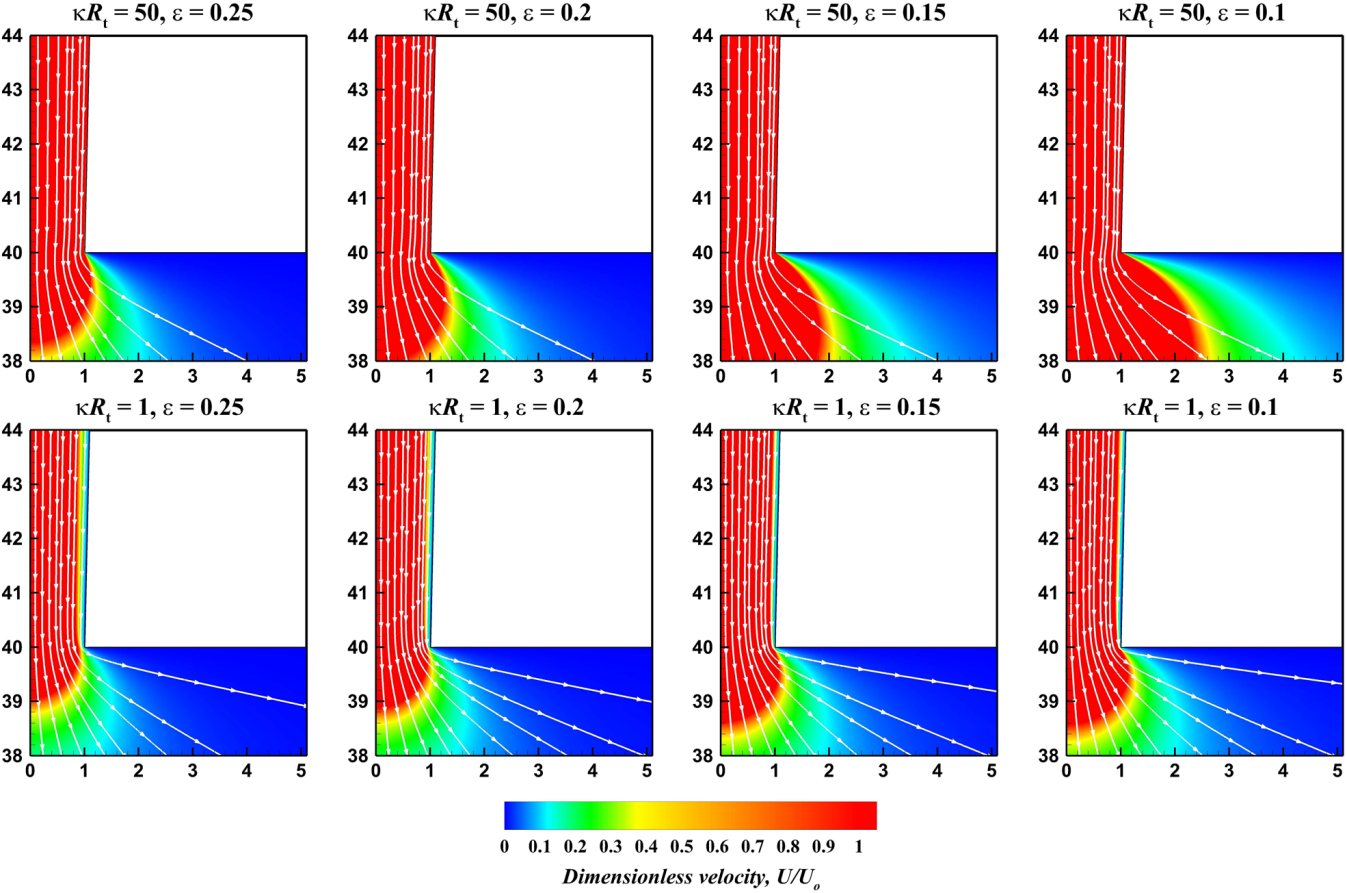
Representative streamlines and velocity contours at $De= 100$, $V=40$, $\sigma = -50$ and extreme values of $\kappa R_t$ and $\varepsilon$.

Dimensionless constitutive equation of the sPTT fluids (i.e., Eqs. 8–10) can be written as follows:

16 $$\begin{aligned} \tau ^{*}+\frac{De}{\kappa {R_t}}(\overset{\nabla }{\tau ^{*}}+\varepsilon {\tau _{kk}}^{*}{\tau }^{*})=2 \mathbf{D ^{*}} \end{aligned}$$

where $u^*$, $p^*$, $\tau ^*$ and $\psi ^*$ are the dimensionless velocity, pressure, the polymeric tensor, and electric potential respectively.

Here *De* is the Deborah number and it is defined as,

17 $$\begin{aligned} De = \lambda U_{o}\kappa \end{aligned}$$

where

18 $$\begin{aligned} \kappa = \frac{1}{\lambda _D} = \left( \frac{2{C_o}e {z^2} F}{\varepsilon _p k_B T}\right) ^{1/2} \end{aligned}$$

which is known as the Debye-Hückel parameter. Here $\lambda _D$ has units of length. The ${R_t}/{\lambda_D}$ or $\kappa R_t$ gives the measure of the thickness of the electric double layer (EDL) developed near the charged pore walls and known as the dimensionless Debye length.

**Figure 6:**
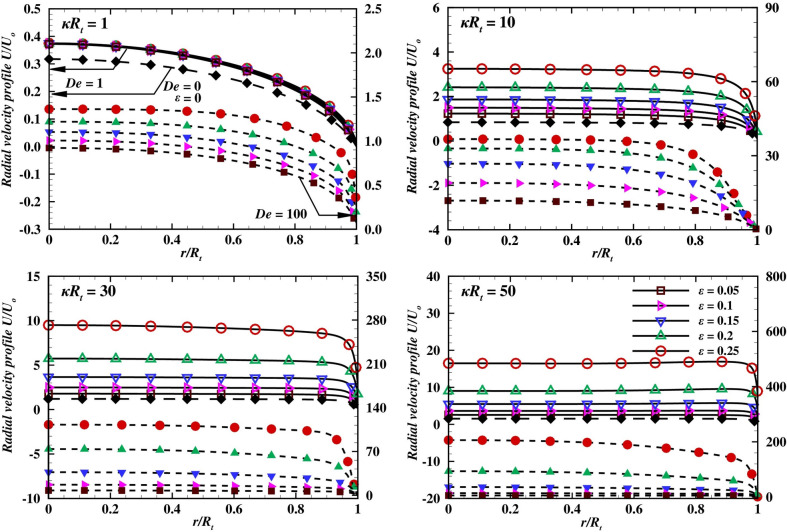
Radial velocity variation with $\varepsilon$ at $De= 1$ (empty symbols) and $De= 100$ (filled symbols), $V=40$, $\sigma = -50$, and Newtonian fluid for various values of $\kappa R_t$.

**Figure 7:**
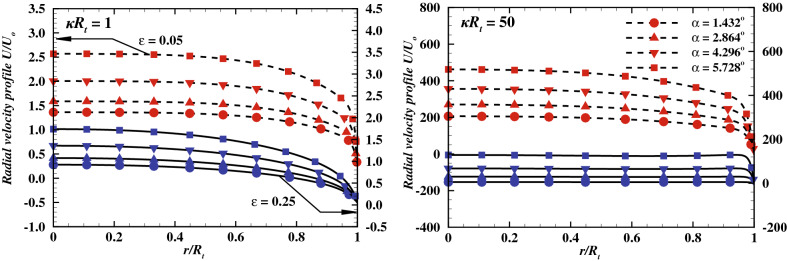
Radial velocity variation with $\alpha$ at $\epsilon$ = 0.05 (red symbols) and $\epsilon$= 0.25 (blue symbols), *V* = 40, *De* = 100 and $\sigma = -50$ and for two extreme values of $\kappa R_t$.

The migration of ions, in turn leads to the flow of electrical current in the nanopore, thus the surface average current across the nanopore is written as follows:

19 $$\begin{aligned} I^*=\frac{I}{I_o}=\frac{1}{{R_t}^2} \int _{S} \left( \sum _{i}^{2} z_{i} {{N}_{i}}^*\right) \cdot {n} \mathrm {d} S \end{aligned}$$

The dimensionless surface charge density is defined as^51^:

20 $$\begin{aligned} \sigma =\frac{\rho _{s}}{ z F {{R_t}^2} C_{o}}=- 2 \sinh \left( \frac{e z}{k_{B} T} \psi _o\right) \end{aligned}$$

The boundary conditions are given below.

- At the boundary ${(1)}$
  A uniform pressure (i.e., $p^* = 0$) with zero stress and fixed potential ${V}^*$ is imposed and ionic concentration is fixed as ’1’.
  $$p^* = 0, \quad {\nabla ^* . u^* = 0},\quad {c_i}^* = 1, \quad \psi ^* = 0.$$
- At the boundary ${(2)}$
  The gradients of potential and concentration are zero and the slip condition for velocity has been imposed.
  $$n \cdot u^* = 0, \quad {\nabla ^* . \psi ^* = 0}, \quad {\nabla ^* . {c_i}^* = 0}.$$
- At the boundary ${(3)}$

**Figure 8:**
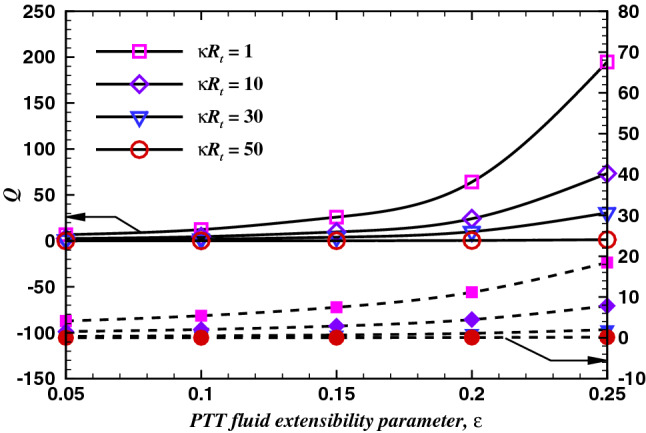
Dimensionless volumetric flow rate variation with $\varepsilon$ for various values of $\kappa R_t$ for $De= 1$ (dashed line) and $De= 100$ (solid line) at $V=40$, and $\sigma = -50$.
  Similarly, the gradients of potential and concentration are zero and no-slip velocity condition at the surface is applied.
  $${u_j}^* = 0, \quad {\nabla ^* . \psi ^* = 0}, \quad {\nabla ^* . {c_i}^* = 0}.$$
- At the boundary ${(4)}$
  The no-slip velocity condition at the surface with zero concentration gradient is considered and a fixed surface charge density $\sigma$ has been imposed, which has been expressed in terms of fixed applied potential $\psi _o$ by Eq. (19).
  $${u_j}^* = 0, \quad {\nabla ^* . \psi ^*} = -\frac{\sigma }{\varepsilon _p}, \quad {\nabla ^* . {c_i}^* = 0}.$$
- At the boundary ${(5)}$
  A uniform pressure (i.e., $p^* = 0$) with zero stress and electric potential is fixed as zero (ground) and ionic concentration is fixed as ’${C_o}^*$’.
  $$p^* = 0, \quad {\nabla ^* . u^* = 0},\quad c_i = 1, \quad \psi ^* = \pm V.$$
- At the boundary ${(6)}$
  For velocity, concentration and potential fields axi-symmetric boundary condition has been imposed.
  $$n \cdot {\nabla ^* . u^* = 0}, \quad n \cdot {\nabla ^* . \psi ^* = 0}, \quad n \cdot {\nabla ^* . {c_i}^* = 0}.$$

## Results and discussion

### Validation

In order to ascertain the accuracy and precision of the numerical results, it is imperative to perform a few benchmark comparisons. Therefore, the chosen numerical scheme (see the supporting information) has been thoroughly scrutinized by comparing the present results with established experimental^19^, analytical^17^, and numerical^18^ studies. A scant number of theoretical findings^16,17,30,40,52,53^ are available on the electro-osmotic flow. The potential profile for a non-convective flow of binary electrolyte (KCl) over a charged surface has been compared with analytical solution (Eq. 21) derived by the Gouy–Chapman theory^17,54^ for two values of bulk ionic concentration, $C_o$ of 1 mM and 100 mM and a fixed surface charge density of $\sigma = -0.001C/m^3$ (see Fig. 2).

21 $$\begin{aligned} \phi (x)=\frac{2 k_B T}{ez} \ln \frac{1-K \exp (-x / {L_o})}{1+K \exp (-x / {L_o})} \end{aligned}$$

**Figure 9:**
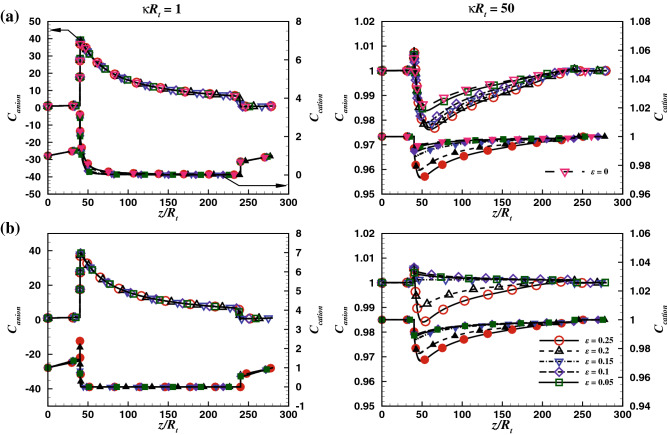
Axial average concentration variation of anion (empty symbols) and cation (filled symbols) with scores of values of $\varepsilon$ including the Newtonian fluids, for $V=40$, $\sigma = -50$ and two extreme values of $\kappa R_t$ (*a*) $De= 1$ and (*b*) $De= 100$.

where *x* represents distance along the charged wall; $K = S /\left[ 2+\left( 4+S^{2}\right) ^{1 / 2}\right]$ and $S = -\lambda ez \sigma /(k_B T \varepsilon )$. The results from the applied numerical schemes are validated with the analytical solutions with an excellent agreement, as demonstrated by Fig. 2. In addition, the present numerical scheme has been validated with experimental (Petrossian et al.^19^) and numerical (Liu et al.^18^) studies, and the corresponding comparisons are presented in Figs. 3 and 4. The present results agree well with the respective experimental results (max. difference 8%, in the case of Petrossian et al.^19^). Thus, the current numerical settings are used to perform extensive numerical simulations, and the resultant velocity, potential, and concentration fields are further derived in terms of velocity contours, streamlines and the velocity profiles, volumetric flow rate, average concentration profiles, and profiles of applied potential with net ionic current passing through the nanopore in terms of the Debye length ($\kappa R_t$), Deborah number (*De*), sPTT extensibility parameter ($\varepsilon$), applied potential difference (*V*) and the surface charge density ($\sigma$).

### Streamlines and the velocity contours

The velocity magnitude contours and streamlines demonstrate the features of the flow, such as the spatial variations in velocity distribution and flow field developments with the varying governing parameters. The electro-osmotic force bears a positive dependence on the bulk concentration. Thus, it is expected that the flow field may intensify with the bulk concentration. Similarly, the applied potential difference also strengthens the volumetric electro-osmotic force^55^, and again it can be speculated that the higher the applied potential difference, the stronger the flow fields. In the EOF, the velocity and stress fields depend upon both the rheological as well as the electrochemical properties of the fluid, i.e., the density, viscosity, the applied electric potential, the surface charge density on the wall, and the bulk ionic concentration. For instance, stability criteria for the flow of viscoelastic Oldroyd-B fluid in a microchannel depends on the bulk electrolyte concentration as well as the applied potential Ji et al.^56^. The further discussion over the flow field in EOF of viscoelastic fluids is limited by a few scarce studies^42,44,45^. These studies include some bounded insights to the viscoelastic flow behavior for flow in microchannel/microtube for few specific sets of governing variables, e.g., bulk concentration, applied potential etc., and the properties and characteristics of viscoelastic EOF have not yet been comprehensively studied for a conical nanopore with a given surface charge.

**Figure 10:**
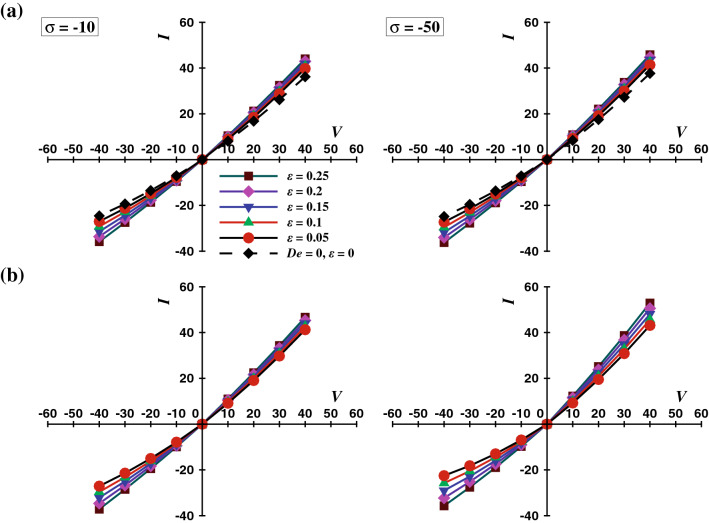
*I* vs *V* curve at different values of $\varepsilon$ and Newtonian fluid at two extreme values of $\sigma$ at **(a)** $De = 1$ and **(b)** $De= 100$ and $\kappa R_t = 50$.

**Figure 11:**
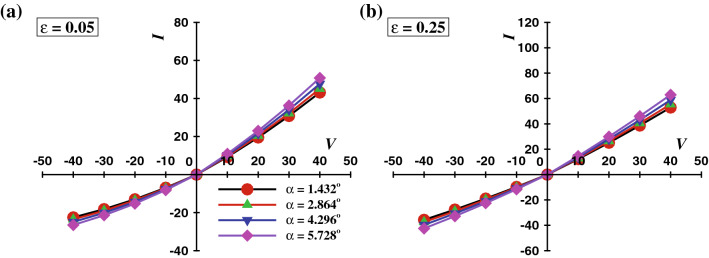
*I* vs *V* curve at different values of $\alpha$ at two extreme values of PTT parameter **(a)** $\varepsilon$ = 0.05, and **(b)** $\varepsilon$ = 0.25 at $\kappa R_t$ = 50, *De* = 100, and $\sigma$ = -50.

Furthermore, the rheological behavior of viscoelastic sPTT fluids depends upon dimensionless relaxation time, *De* and extensibility parameter, $\varepsilon$^49,50^. Therefore, it is important to inspect and investigate the streamlines and velocity contours to visualize the developments in the flow field with the varying governing parameters. Figure S1 (see the supporting information) and Fig. 5 present the streamlines and the dimensionless velocity contours considering two extreme values of $\kappa R_t$ and different values of $\varepsilon$ for $V = 40$, $\sigma = -50$ and $De = 1$ and $De = 100$ respectively. It can be deduced from these plots that in EOF with smaller relaxation time, i.e., $De = 1$ (Fig. S1), and for thin Debye length, i.e., $\kappa {R_t} = 50$, the effective fluid viscosity near the nanopore wall diminishes as the fluid extensibility ($\varepsilon$) increases. Thus, the magnitude of the velocity field strengthens with the increase in the value of $\varepsilon$. In other words, fluid extensibility decreases the effective viscosity, which mimics the so-called shear-thinning nature of the fluid. Therefore, it is concluded that the overall velocity magnitude increases with the extensibility of viscoelastic (sPTT) fluids. At the highest Debye length (i.e., $\kappa {R_t} = 1$), a thick electric double layer forms adjacent to the wall. This electric double layer overlaps and engulfs the flow area at the convergent end of the nanopore (i.e. nanopore tip), and thus, the flow is expected to be constricted. Therefore, the velocity magnitude near the walls is observed to be weaker than that of the core of the flow. The overlap of the electric double layer gives rise to a low shear region, and therefore the influence of fluid extensibility ($\varepsilon$) on the flow field is observed to be negligible in this limit. In other words, the $\varepsilon$ has little to no influence on the flow field at such low values of $\kappa R_t$ (i.e., $\kappa R_t$ = 1) and *De* (i.e., $De = 1$) at the nanopore tip. Essentially, the rheological parameters i.e. the Deborah number *De*, the sPTT extensibility parameter $\varepsilon$ and the Debye length $\kappa R_t$ exhibit a positive influence over the spatial flow distribution.

### Volumetric flow rate: effect of *De*, $\varepsilon$ and $\kappa R_t$

From the discussion of the velocity field contours inside the conical nanopore presented in the previous section, it could be inferred that, the overlapping of the electric double layer at the convergent end (the tip) of the nanopore affects the momentum flux through the nanopore^45,52,57^. The insights about the characteristics of the flow field in EOF for a micro-channel/micro or nanopore can also be visualized by the corresponding radial velocity profiles as presented in Figs. 6 and 7. The velocity profiles in a 2D micro-geometry (micro-channel/nozzle) has been investigated in terms of the Debye length, $\kappa R_t$, Deborah number, *De*, cone angle, $\alpha$ and rheological constraints by several numerical studies, such as Bezerra et al.^58^, Chen et al.^45^, Mei et al.^44^, and Tseng et al.^47^ as well as analytical studies e.g. Afonso et al.^52,57^ and Wang et al.^43^.

**Figure 12:**
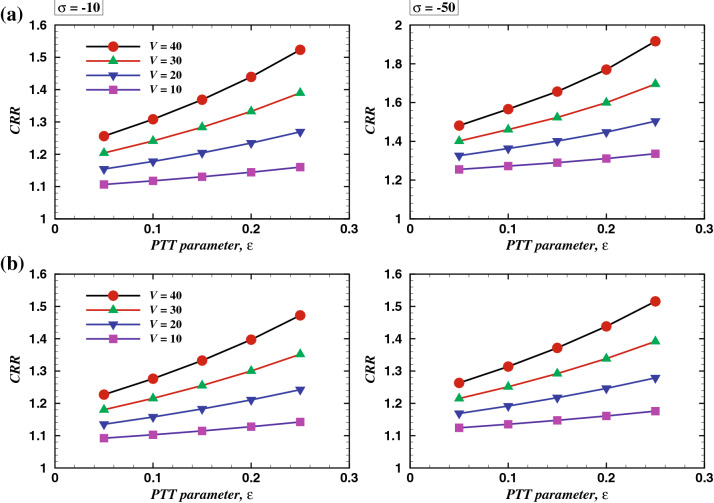
Variation of current rectification ratio (*CRR*) with $\varepsilon$ for two values of $\sigma$ and $\kappa R_t$ = 50 at (*a*) $De= 1$ and (*b*) $De= 100$.

As discussed in previous section, the Debye parameter, $\kappa R_t$ and Deborah number, *De* exhibit a positive influence on the flow field. In other words, an increase in $\kappa R_t$ and *De* increases the average and maximum velocity. Therefore, the radial velocity profiles at the tip of the nanopore are shown in Fig. 6, for $V_o = 40$, $\sigma = -50$, four different values of Debye length $\kappa R_t$, and various values of $\varepsilon$ with two extreme values of the Deborah number i.e., $De = 1$ and $De = 100$, respectively. Moreover, results of Newtonian fluids are also added for the sake of comparison. The trends in Fig. 6 can be illustrated as follows: the velocity profile of Newtonian fluids has been found to be a lower than that of a visco-elastic fluid. The difference between the maximum velocity profile of the Newtonian fluid and for the fluid with the lowest relaxation time and PTT parameter is found to be proportional to the Debye length and varies roughly from 20% to 700% as Debye length is varied from highest value (i.e. $\kappa R_t$ = 1) to the lowest value ($\kappa R_t$ = 50). Furthermore moving towards the purely visco-elastic behaviour, as for the lowest Deborah number, i.e., $De = 1$ and for the highest Debye length ($\kappa R_t = 1$), the velocity profile corresponds to the different values of $\varepsilon$ overlap to each other at the nanopore tip. This effect suggests that the fluid extensibility ($\varepsilon$) has a negligible effect on the velocity field. Upon gradual reduction in Debye length, the value of the parameter $\kappa R_t$ increases. The maximum velocity shows a positive dependence on the value of $\kappa R_t$, for a given value of $\varepsilon$. In brief, the lower the thickness of the electric double layer, the higher the maximum velocity. In addition, the influence of the fluid extensibility on $\kappa R_t$ ($\varepsilon$) also reflects in the flow field and the maximum velocity, it increases by $\approx 170\%$ for $\kappa R_t = 10$ and by $\approx 470\%$ for $\kappa R_t = 50$ as the value of $\varepsilon$ varies from 0.05 to 0.25. This clearly suggests a significant influence of the value of $\varepsilon$ on the maximum velocity at a smaller Debye length. For a fixed value of $\varepsilon$, the velocity distribution across the nanopore gradually shifts from a parabolic-like profile to the plug-like profile upon increasing *De* (Fig. 5) and $\kappa R_t$ as shown in Fig. 6. Clearly, the proportion of the solid-like behavior increases with the Deborah number, and thus the flow at the center of the nanopore behaves like a plug while the fluid-like response is seen towards the wall with a steep velocity gradient. Similarly, the thinner electric double layer also gives rise to a plug-like velocity profile, irrespective of Deborah number. This is simply because of the low flow resistance for the migration of ions outside the electric double layer. At $De = 100$, the effect of $\varepsilon$^45^ on the resultant velocity profiles is significant even at $\kappa R_t = 1$. Furthermore, the maximum velocity exhibits a similar dependence on the values of $\varepsilon$ and $\kappa R_t$, as illustrated before. Precisely, the maximum velocity is observed to enhance with the value of $\varepsilon$ by $\approx 300\%$ for $\kappa R_t = 10$ and by $\approx 2000\%$ for $\kappa R_t = 50$. All in all, it has been observed that the momentum flux near the nanopore wall enhances more profoundly with the PTT parameter, $\varepsilon$ than with the variation of the Debye parameter, $\kappa R_t$. In summary, it is concluded that the fluid extensibility ($\varepsilon$) and the Deborah number (*De*) strengthen the flow field (Fig. 5), while the value of $\kappa R_t$ and $\sigma$ controls the thickness of the electric double layer in the nanopore. Furthermore, It has been reported that the average velocity varies linearly with the applied electric potential and exhibits an inverse dependence on the polymeric viscosity^56^. Furthermore, in a conical nanopore, an increase in the cone angle accompanies the increase in the associated flow area. Also, the body force exerted on the fluid by the applied potential bias is proportional to the flow area. Thereby, suggesting the corresponding trends of velocity, concentration and ionic current profiles with respect to the variation in the cone angle. Tseng et al.^47^ have discussed the effect of the cone angle on the velocity as well as the concentration fields.They have demonstrated that, for a fixed charge density and the applied voltage, as the cone angle increases, the velocity field and ionic current within the nanopore intensifies whereas the average cation concentration depletes. Therefore, the variation in the cone angle has been analysed for four successively increasing values of $\alpha$, starting from $1.432^o$ and upto $5.728^\circ$. Figure 7 presents the radial velocity profile variation with the cone angle $\alpha$, for a fixed applied potential bias ($V = 40$) and surface charge density ($\sigma$ = -50), the maximum Deborah number ($De = 100$), and two extreme values of the Debye length and PTT parameter. The maximum velocity and therefore the average volumetric flow rate is found to be positively dependent on the cone angle. As the cone angle, $\alpha$ increases from $1.432^\circ$ to $5.728^\circ$, for lowest value of $\kappa R_t$ or the highest Debye length, the corresponding results show an 88.46% increase in the maximum velocity for $\varepsilon$ = 0.05 and an 91.23% increase for $\varepsilon$ = 0.25. When the Debye length is minimum and $\kappa R_t$ = 50, the maximum velocity increases by 138.45% for $\varepsilon$ = 0.05 and 197.65% for the highest fluid extensibility i.e. $\varepsilon$ = 0.25. Thus it could be concluded that the maximum velocity is directly dependent on the cone angle and the highest variation is achieved at the maximum fluid extensibility or the highest value of PTT parameter and the lowest value of Debye length or the highest value of $\kappa R_t$. Moreover, the average velocity can also be quantified in terms of volumetric flow rate. Therefore, the variation of the dimensionless volumetric flow rate with the values of $\varepsilon$ and for various values of $\kappa R_t$ at $V_o = 40$, $\sigma = -50$, $De = 1$ and $De = 100$, is shown by Fig. 8.

**Figure 13:**
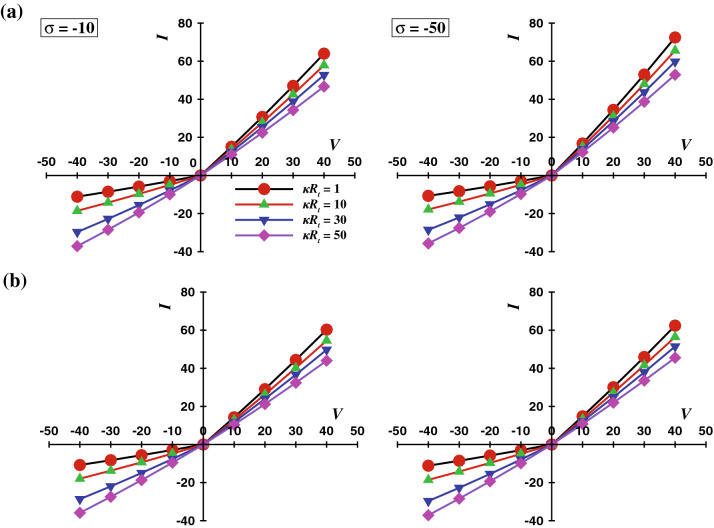
*I* vs *V* curve at different values of $\kappa R_t$ at *De* = 100 **(a)** $\varepsilon = 0.05$ and **(b)** $\varepsilon = 0.25$.

Similar to the discussion given for the radial velocity profiles, the dimensionless volumetric flow rate, *Q* is found to positively correlate with the value of the $\kappa R_t$ and *De*. Also, the volumetric flow rate monotonically increases with the value of $\varepsilon$. Furthermore, the increase in the flow rate is observed to be more pronounced at $De = 100$ than that at $De = 1$, at $\varepsilon > = 0.17$. In summary, the volumetric flow rate shows a positive correlation with the $\kappa R_t$, *De*, and $\varepsilon$.These trends are in line with results reported for the straight channel by Ji et al.^56^. It is also to be noted that the average velocity varies linearly with the applied electric potential and exhibits an inverse dependence on the polymeric viscosity.

### Concentration distribution: effect of $\kappa R_t$, *De* and $\varepsilon$

The axial concentration distribution provides useful insights into the EOF phenomena in a conical shape nanopore. In the absence of elastic effects (i.e., $De=0$)^16,18,22,23,55^ increasing the applied potential increases the concentration of ions near the nanopore tip. The maximum in the concentration profile occurs near the nanopore tip, and it increases with the increasing electro-negativity of the cation of salt. The pH of the medium also exhibits an aiding effect on the concentration distribution. Figure 9, represent the variation of the average ionic concentration for the anions and cations along the length of nanopore, for two values of Debye length parameter (i.e., $\kappa R_t = 1$ and 50), the extreme values of Deborah number (*De*), $V = 40$ and for a fixed surface charge density $\sigma = -50$. The results corresponding to the Newtonian fluids have also been added for the sake of comparison in the enhancement in the concentration distribution by the viscoelastic fluids over the Newtonian fluids. It has been observed that at the Highest value of Debye length (i.e. $\kappa R_t$), difference between the concentration profiles of Newtonian and the visco elastic fluid is almost negligible, thus all the plots appear to merge into one curve. Whereas, an augmentation of 16% has been observed for the Newtonian fluid for $\kappa R_t$ = 50 in comparison with the visco-elastic fluids. Since an electric double layer exists near the negatively charged pore surface, cations in the electrolyte migrate under the electrostatic force due to the charged nanopore wall. In contrast, the anions migrate away from the wall and merge with the bulk flow towards the nanopore tip. Therefore, the average anion concentration is found to be maximum near the tip of the nanopore and gradually decreases towards the base of the nanopore. Since the radius of the base end of the nanopore is significantly larger than the Debye length (i.e., $\kappa R_t$), thus the local electric double layer does not interact at the base. The overlapping phenomenon of the double layer is only relevant at the convergent end of the nanopore (i.e., the tip of the nanopore). At $De = 1$ (see Fig. 9a), for $\kappa R_t = 1$, the value of $\varepsilon$ has no effect on the the dimensionless anion or cation concentration profiles. On the other hand, at $\kappa R_t = 50$, which mimics the lowest Debye length, the flow resistance is expected to be much smaller than that at $\kappa R_t = 1$. At $De = 1$, the migration of anion and cation towards the surface of the nanopore and the nanopore core is facilitated by the EOF and found to be moderately influenced as the value of $\varepsilon$ increases. Also, the effect of the extensibility parameter (i.e., $\varepsilon$) is significantly visible at $De = 100$ (Fig. 9b), since there are much sharper velocity gradients near the wall compared to the case at $De = 1$. Precisely, the ionic concentration distribution of both cations and anions is found to be maximum at the tip of the nanopore and decline towards the nanopore base. Moreover, the ionic concentration distribution is found to be invariant with the $\varepsilon$ and *De* at $\kappa R_t = 1$. While variations with $\varepsilon$ are observed at $\kappa R_t = 100$. The concentration profile flattens with an increase in the value of $\varepsilon$. On the other hand, *De* exhibits an inverse dependence on the concentration distribution at $\kappa R_t = 50$. In a nutshell, it has been found that at high values of Debye length, i.e., $\kappa R_t = 1$, relatively lower concentration gradients observed at the tip of the conical nanopore for both the anions and cations, indicating the retarding of the ionic mass transport due to the interacting electric double layer at the tip of the nanopore. On the other hand, decreasing the Debye length sharpens the ionic concentration gradients indicating the strong ionic current flow. In addition, the rate of ion transport also shows a positive dependence on Deborah number. Therefore, it can be deduced that the ionic mass flux is significantly dependent upon the Debye length $\kappa R_t$, fluid extensibility $\varepsilon$ and the Deborah number *De*.

### Viscoelastic effect on *ICR* and *CRR*

The total ionic flux in EOF constitutes three components: the convective, the diffusive, and the electro-osmotic flux, respectively. The total ionic flux is integrated across the area of the nanopore tip and quantified as the total ionic current through a nanopore. The corresponding definition is expressed by Eq. 18. To the date^16,18,22,23,55^ the ICR phenomena and its characteristics are investigated in terms of the governing parameters such as the bulk ionic concentration^15,16,18,23,59^, $C_o$, the surface charge density^22,30^, $\sigma$, salt type^55^ and the pH of the electrolyte^11^. All in all, it is reported that the ICR phenomena accentuate with the increasing value of pH, $\sigma$, $C_o$, and the electronegativity of the salt cation. Here we have demonstrated the individual and/or combined effects of the Deborah number *De*, the PTT parameter $\varepsilon$, the Debye length $\kappa R_t$ and the surface charge density $\sigma$ on the ICR phenomena, the total ionic current through the nanopore has been plotted against the corresponding applied potential bias *V* as *I* vs *V* graphs. Figure 10 shows the current vs potential relationship for two extreme values of the Deborah number ($De = 1$ and 100), and two values of surface charge density ($\sigma$ = -10 and -50), at a fixed Debye length ($\kappa R_t = 50$) and for scores of values of $\varepsilon$. These figures also incorporate the ionic current values for Newtonian fluids and it has been observed that Newtonian fluids correspond to the lowest ionic current in the nanopore and the ionic current shows the least rectification (ICR) in the case of Newtonian fluids. The ionic current is found to vary with the applied potential monotonically. The ionic current increases with the value of $\varepsilon$. At a low Deborah number (i.e., $De = 1$) (Fig. 10a), the surface charge density, $\sigma$ has a negligible influence on the ionic current. Thus it is concluded that $\sigma$ does not have a significant influence on the ICR at $De = 1$. While, as the value of *De* increases, it intensifies the flow rate, which, in turn, augments the rate of total ionic flux. Moreover, the surface charge density, $\sigma$ demonstrates a positive dependence on the ICR phenomena at a higher Deborah number ($De = 100$) (Fig. 10b). This effect is similar to the reported results on ICR in the absence of elastic effects. Figure 11 demonstrates the corresponding relationship of the ionic currant and the applied potential for a fixed surface charge density $\sigma$ = -50, *De* = 100 and $\kappa R_t$ = 50 for two extreme values of fluid extensibility. It can be inferred from the results that the value of ionic current as well as the rectification (ICR) is maximum for the maximum value of cone angle. Moreover the fluid extensibility facilitates the ionic current at the maximum value of cone angle. With the increasing value of $\alpha$, the ICR is shown to be more pronounced at $\varepsilon$ = 0.25 than at $\varepsilon$ = 0.05, suggesting that the PTT parameter has a significant influence on ionic current and the rectification phenomena at elevated cone angle. Figure 12 shows the variation of the current rectification ratio (CRR)^22^ with the extensibility parameter ($\varepsilon$). The CRR is defined as the ratio of the value of ionic current for the forward and backward potential bias of the same magnitude. The resulting trends are observed to agree with the previous discussion on the velocity field. The CRR demonstrates a positive dependence on the extensibility parameter (i.e., $\varepsilon$), the Deborah number, *De*, the surface charge density, $\sigma$, and the applied potential bias *V*. In addition, an increase of 30% in the CRR value has been observed as the $\varepsilon$ varies from its lowest to its highest value for a maximum value of *V*, *De* and $\sigma$. Figure 13 and Figure S2 demonstrate the *I*–*V* curves to delineate the effect of $\kappa R_t$ and *De* respectively. Figure 13 shows the influence of the Debye length ($\kappa R_t$) on the ICR behavior of the nanopore, for two extreme values of the value of $\varepsilon$ and the value of $\sigma$ and a given fixed value of Deborah number $De = 100$. Evidently, the ionic current rectification is facilitated by the decreasing value of $\kappa R_t$ or the increasing value of the Debye length. As noted earlier, at a low value of $\kappa R_t$, overlapping of the electric double layers occurs at the tip of the conical nanopore. Thus, it can be deduced that the ICR is higher for the lower values of $\kappa R_t$ (Fig. 13a). As it can be observed from Figure S2 (see the supporting information), the effect of the *De* is only found to be significant for $\varepsilon = 0.25$ and $\sigma = -50$ (Fig. S2b) for a given value of $\kappa R_t = 50$. The ICR is found to be enhanced with the increasing values of $\varepsilon$ and $\sigma$.

## Conclusions

The flow of viscoelastic sPTT fluids through a conical nanopore has been numerically analyzed for following ranges of conditions: $1\le De \le 100$, $1\le \kappa R \le 50$, $0.05\le \varepsilon \le 0.25$, $-40\le V \le 40$, and $\sigma = -10$ and $-50$. The P-N-P model has been used to couple the velocity, concentration, and potential fields, and the numerical results have been delineated in terms of streamlines and velocity contours, radial velocity profiles, volumetric flow rate, surface averaged concentration profiles, and ionic current with the applied potential and current rectification ratio plots. The following conclusions are derived from the present results.

- At a low value of $\kappa R_t$ (i.e.,$\kappa R_t = 1$ ), the Debye length is comparable to the radius of the nanopore tip, and the electric double layer overlaps at the tip of the nanopore. Under these conditions, the electrostatic force between ions and the charged surface becomes stronger than the electro-osmotic force. Therefore, fluid rheology has little to no influence on the flow field, the concentration, and the potential field.
- The flow extensibility parameter exhibits a positive dependence on the velocity, concentration, and potential field. The flow extensibility parameter offers a shear-thinning-like behavior where the effective viscosity decreases with the increased flow extensibility parameter. This, in turn, leads to enhanced momentum and ion transport across the nanopore in comparison to the respective Newtonian fluid behaviour.
- Deborah number (*De*) increases the contribution of solid-like behavior. Thus, the plug-like velocity profile across the nanopore increases with the Deborah number accompanied by high-velocity gradients near the nanopore wall.
- The ionic current through the nanopore is found to be proportional to extensibility parameter ($\varepsilon$), surface charge density ($\sigma$), the Deborah number (*De*) and applied potential bias *V*, while it exhibits an inverse dependence on the $\kappa R_t$, Such inverse trend is attributed to the fact that at high values of $\kappa R_t$ there is no overlap of the electric double layer at the tip of the nanopore. Moreover, the cone angle ($\alpha$) also exhibits a positive influence over the radial velocity and values of ionic current.
- The CRR is the measure of the extent of current rectification. It has been found to increase with the increase in PTT parameter (i.e., $\varepsilon$), surface charge density, the Deborah number, and the applied potential.

## Supplementary Information

Supplementary Information.
