# Supplementary material for: Ion transport and current rectification in a charged conical nanopore filled with viscoelastic fluids
Source: Sci Rep. 2022 Feb 15;12:2547. doi: 10.1038/s41598-022-06079-w (PMC8847403; doi:10.1038/s41598-022-06079-w)
Supplement: Supplementary file 1 — Supplementary Information. [file 41598_2022_6079_MOESM1_ESM.pdf]

Supplementary information

Ion transport and current rectification in a charged conical nanopore filled with viscoelastic fluids

Mohit Trivedi[a],and Neelkanth Nirmalkar[a*]

[a]Department of Chemical Engineering, Indian Institute of Technology, Ropar 140001, India

*Corresponding author: n.nirmalkar@iitrpr.ac.in

Numerical solution scheme:

A finite volume method based solver OpenFOAM in conjunction with the RheoTool toolbox (version 5)(Weller *et al.*, 1998) has been used to solve the governing and constitutive equations (i.e., Eq. 14-15 and 22) with conjunction for the electric potential and the velocity field. PTT model has been used for the constitutive relation. A fully developed solution is considered with the bulk concentration $C_o = 1\ mM$. Diffusities of cations and anions are taken as $9.31 \times 10^{-9}\ m^2 \cdot s^{-1}$ and $5.30 \times 10^{-9}\ m^2 \cdot s^{-1}$. The temperature has been taken as $T = 300\ K$, density $\rho = 10^3\ kg \cdot m^{-3}$, relative permeability $\varepsilon_p = 7.08 \times 10^{-10}\ F \cdot m^{-1}$ and polymeric viscosity $\eta_p = 10^{-3}\ Pa \cdot s$

The momentum and P-N-P equations with the corresponding boundary conditions are solved using OpenFOAM solver (Weller *et al.*, 1998) (version 7) in conjunction with the RheoTool toolbox (version 4.1). The divergence of the velocity, electric potential, polymeric, and conformation tensors has been calculated using Convergent and Universally Bounded Interpolation Scheme for Treatment of Advection scheme (CUBISTA). A second-order accurate Gauss linear orthogonal interpolation scheme and Gauss linear interpolation schemes have been used to solve diffusive and convective terms for velocity, pressure, concentration, and potential fields. The resulting linear system of equations for the velocity, pressure, concentration, and electric fields are solved using the preconditioned bi-conjugate gradient solver (PBiCG). The velocity, pressure, concentration, and potential fields are coupled by the SIMPLE algorithm. The relative convergence criteria of the velocity, concentration, electric potential, and pressure fields have been set as 10^{-10}. The grid independence study has been performed to assure the reliability and reproducibility of the results. Three meshes $G1$, $G2$ and $G3$ of progressively increasing number of domain elements, i.e., $36,500$, $71,200$ and $1,44,600$ respectively (See table S1) have been generated, and resultant ionic current I has been compared for the critical values of the flow, and electro-kinetic parameters and the $G2$ has been found to be optimum for the current study.

Table S1. Grid-independence test at $V_o = 40$.

			Ionic current, I							
			$\sigma = -10$				$\sigma = -50$			
			$\varepsilon = 0.05$		$\varepsilon = 0.25$		$\varepsilon = 0.05$		$\varepsilon = 0.25$	
Grid	Surface elements	Total elements	$De = 1$	$De = 100$	$De = 1$	$De = 100$	$De = 1$	$De = 100$	$De = 1$	$De = 100$
G1	570	36,500	38.4571	40.3128	42.7371	45.3646	39.8690	41.7530	43.8821	51.5191
G2	950	71,200	39.7695	41.2618	44.0135	46.6714	41.1614	43.1422	45.5540	52.8781
G3	1820	1,44,600	40.0674	41.6113	44.3093	46.9533	41.4475	43.5162	45.9931	53.3048

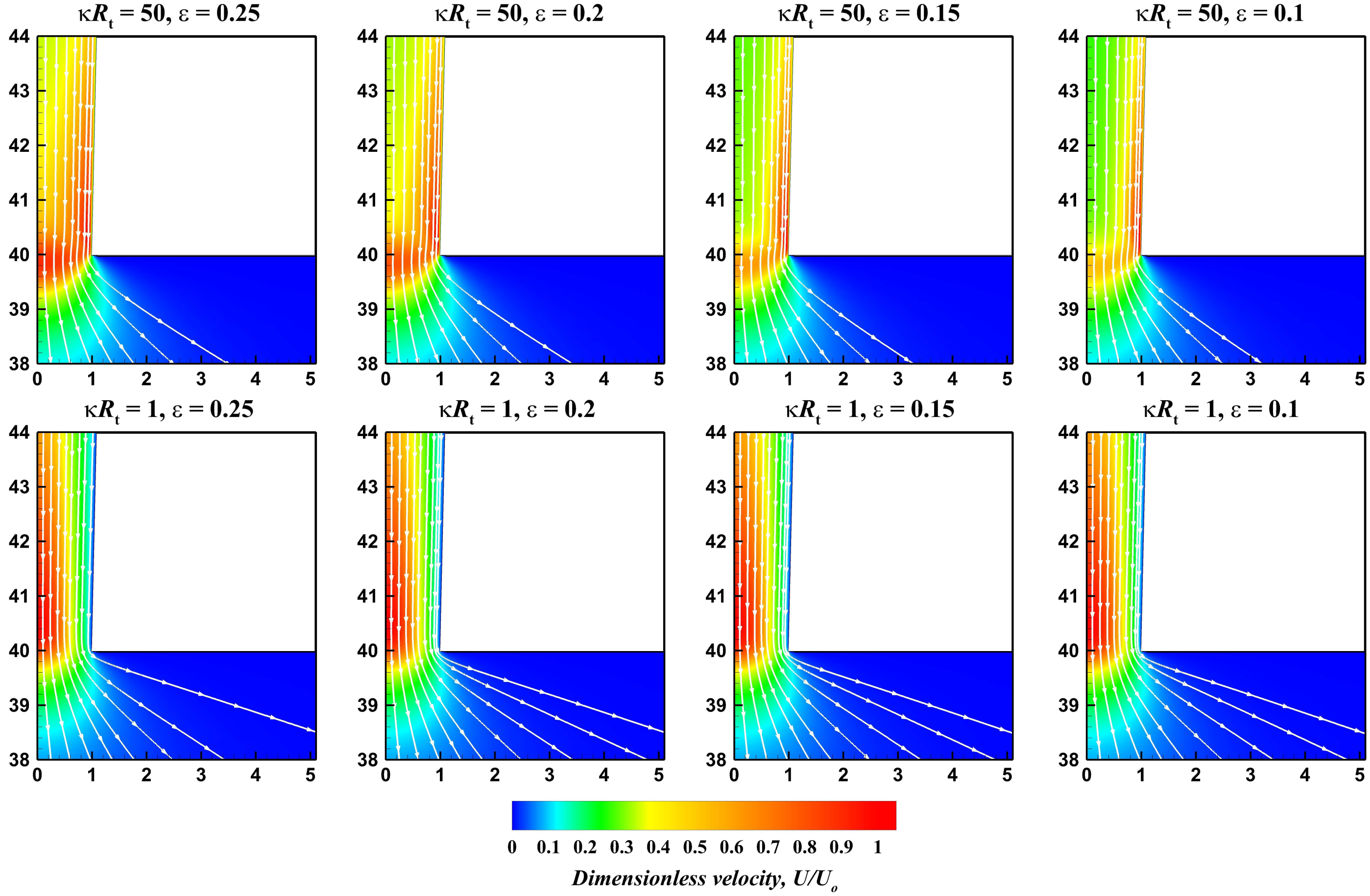

Figure S1. Representative streamlines and velocity contours at $De = 1$, $V_o = 40$, and $\sigma = -50$ and extreme values of κR_t and ε.

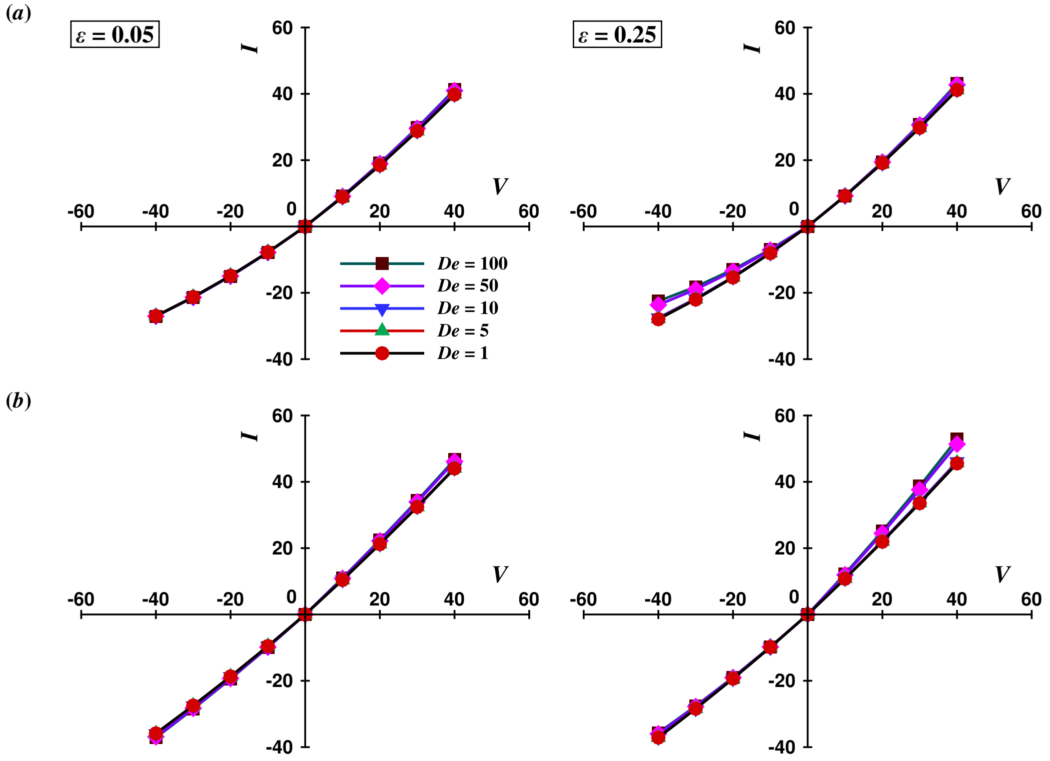

Figure S2. I vs V curve at different values of De at two extreme values of ε and κR_t = 50 at (a) σ = -10 and (b) σ = -50.
